# KAKU4 regulates leaf senescence through modulation of H3K27me3 deposition in the *Arabidopsis* genome

**DOI:** 10.1186/s12870-024-04860-9

**Published:** 2024-03-07

**Authors:** Yaxin Cao, Hengyu Yan, Minghao Sheng, Yue Liu, Xinyue Yu, Zhongqiu Li, Wenying Xu, Zhen Su

**Affiliations:** State Key Laboratory of Plant Environmental Resilience, College of Biological Sciences, China Agricultural University, Beijing, 100193 China

**Keywords:** *Arabidopsis*, H3K27me3, KAKU4, Leaf senescence

## Abstract

Lamins are the major components of the nuclear lamina, which regulate chromatin structure and gene expression. KAKU4 is a unique nuclear lamina component in the nuclear periphery, modulates nuclear shape and size in Arabidopsis. The knowledge about the regulatory role of KAKU4 in leaf development remains limited. Here we found that knockdown of *KAKU4* resulted in an accelerated leaf senescence phenotype, with elevated levels of H_2_O_2_ and hormones, particularly SA, JA, and ABA. Our results demonstrated the importance of KAKU4 as a potential negative regulator in age-triggered leaf senescence in Arabidopsis. Furthermore, we conducted combination analyses of transcriptomic and epigenomic data for the *kaku4* mutant and WT leaves. The knockdown of *KAKU4* lowered H3K27me3 deposition in the up-regulated genes associated with hormone pathways, programmed cell death, and leaf senescence, including *SARD1*, *SAG113/HAI1*, *PR2*, and so forth. In addition, we found the functional crosstalks between KAKU4 and its associated proteins (CRWN1/4, PNET2, GBPL3, etc.) through comparing multiple transcriptome datasets. Overall, our results indicated that KAKU4 may inhibit the expression of a series of genes related to hormone signals and H_2_O_2_ metabolism by affecting the deposition of H3K27me3, thereby suppressing leaf senescence.

**Supplementary Information:**

The online version contains supplementary material available at 10.1186/s12870-024-04860-9.

## Running title

Knockdown of *KAKU4* speeds leaf senescence.

**Summary Statement**:

In summary, our results demonstrated the importance of KAKU4 as a potential negative regulator in age-triggered leaf senescence through modulating H3K27me3 deposition in Arabidopsis. This work may advance the knowledge about the regulatory role of plant nuclear lamina proteins or the regulation of leaf senescence.

## Introduction

The nuclear lamina (NL) is an important structural determinant for the nuclear envelope, covering the inner surface of the inner nuclear membrane (INM). Lamina consists of a thin meshwork of lamins, which are structural components of the nuclear lamina that regulate genome organization and gene expression [1–5]. A point mutation in the eleventh exon of the lamin encoding gene LMNA can cause Hutchinson-Gilford progeria syndrome (HGPS), a rare premature-aging disease in children. This mutation affects the process of alternative splicing, resulting in the accumulation of the truncated abnormal protein, which leads to a series of changes in the nucleus, including in nuclear morphology, DNA repair, chromatin remodeling, and nuclear transport [6–8]. Lamin A buffers Casein kinase 2 (CK2) activity to modulate aging in a progeria mouse model [9]. Declining lamin B1 expression mediates age-dependent decreases of hippocampal stem cell activity [10]. Thus, the defects in the components of the nuclear lamina can affect the morphology of the nucleus, leading to human diseases and aging.

Although no lamin-homologous proteins have been found in plants, the putative lamina-like structures have been discovered in the carrot, and a nuclear matrix constituent protein (NMCP1) has been identified as a peripheral framework component of its nuclei [11]. In Arabidopsis, multiple lamin-like proteins are identified, including CRWNs [12, 13], KAKU4 [14], NEAPs [15], and PNET2 [16]. Especially, the proteomics technologies speed up to identify the complex components among the interaction partners of CRWN1, KAKU4, PNET2_A/B, NEAP1, the nuclear basket protein GBPL3, and some other lamina related components [16–19].

Four CRWNs, named CRWN1, CRWN2, CRWN3, and CRWN4, are NMCP homologs in Arabidopsis [13, 20]. The mutants *crwn1* and *crwn4* show aberrantly small and spherical nuclei in the epidermal cells of the leaf and root [12]. In addition to nuclear morphology and size, CRWNs are involved in the regulation of a series of biological processes. The double mutant *crwn1crwn2* and *crwn1crwn4* exhibit ectopic defense responses and spontaneous cell death against the virulent bacterial pathogens, which is related to the upregulation of salicylic acid (SA)-biosynthesis gene *SID2* and increase SA content [21]. CRWNs play an important role in diminishing ROS accumulation and protecting genomic DNA against excessive oxidative damage induced by the genotoxic agent methyl methanesulfonate. Under normal growth conditions, *crwn1crwn3*, *crwn2crwn3*, and *crwn2crwn4* show enhanced leaf cell death, over-accumulation of ROS, and slightly earlier leaf senescence [22].

Meanwhile, CRWN1 is involved in maintaining chromatin compartmentalization [23]. The loss of *CRWN1* results in the loss of specific chromatin distribution at the nuclear periphery. Furthermore, CRWN1, CRWN4, and non-CG DNA methylation have recently been discovered to be involved in regulating chromatin tethering at the nuclear periphery [24]. CRWN1 interacts with the copper-associated gene locus, localized to the nuclear lamina under excess copper conditions [25]. In addition, PWWP-domain interactor of polycombs (PWO1) has been reported to physically interact with CRWN1 protein, functioning in the same pathway to affect nuclear morphology. Further, it regulates the expression of a similar set of targets, which have significant overlap with H3K27me3 targets [26]. PWO1 is involved in polycomb group (PcG) silencing by repressing H3K27me3 accumulation in a subset of PcG targets [27]. Polycomb repressive complex 2 (PRC2) can trimethylate lysine 27 on histone H3 (H3K27me3) [28]. H3K27me3 accumulation levels near genes involved in salicylic acid biosynthesis are decreased in *crwn* mutants [29]. Recently, AtGBPL3/CRWN1&4/KAKU4 complex has been identified to play an essential role during interphase in H3K27me3-associated transcriptional repression at the nuclear periphery [30].

KAKU4 protein has been identified as a nuclear inner envelope protein and a unique nuclear lamina component in the nuclear periphery, physically interacts with CRWN1 and CRWN4, and modulates nuclear shape and size [14]. Similar to *crwn1* and *crwn4*, the nuclei of the *kaku4* mutant in epidermal cells of cotyledons, hypocotyls, and roots, are smaller and less elongated than those of wild-type (WT) plants. KAKU4 is mediated by the deformation of the vegetative nucleus of pollen grains and controls its migration over sperm cells in pollen tubes [31]. However, the knowledge of the biological functions and gene regulation network of KAKU4 protein remains limited. In this study, we found that the knockdown of the *KAKU4* gene resulted in significant early leaf senescence. In the *kaku4* mutant, H_2_O_2_ was obviously accumulated, and the plant hormones salicylic acid (SA), jasmonic acid (JA), and abscisic acid (ABA) were also increased. To elucidate the possible molecular mechanisms underlying the KAKU4 protein regulating plant leaf senescence, we conducted high-throughput sequencing technology, including RNA-seq and ChIP-seq data analyses. Our results suggested that KAKU4 may modulate H3K27me3 deposition to regulate leaf senescence in Arabidopsis.

## Results

### Knockdown of *KAKU4* results in an accelerated leaf-senescence phenotype

**Fig. 1:**
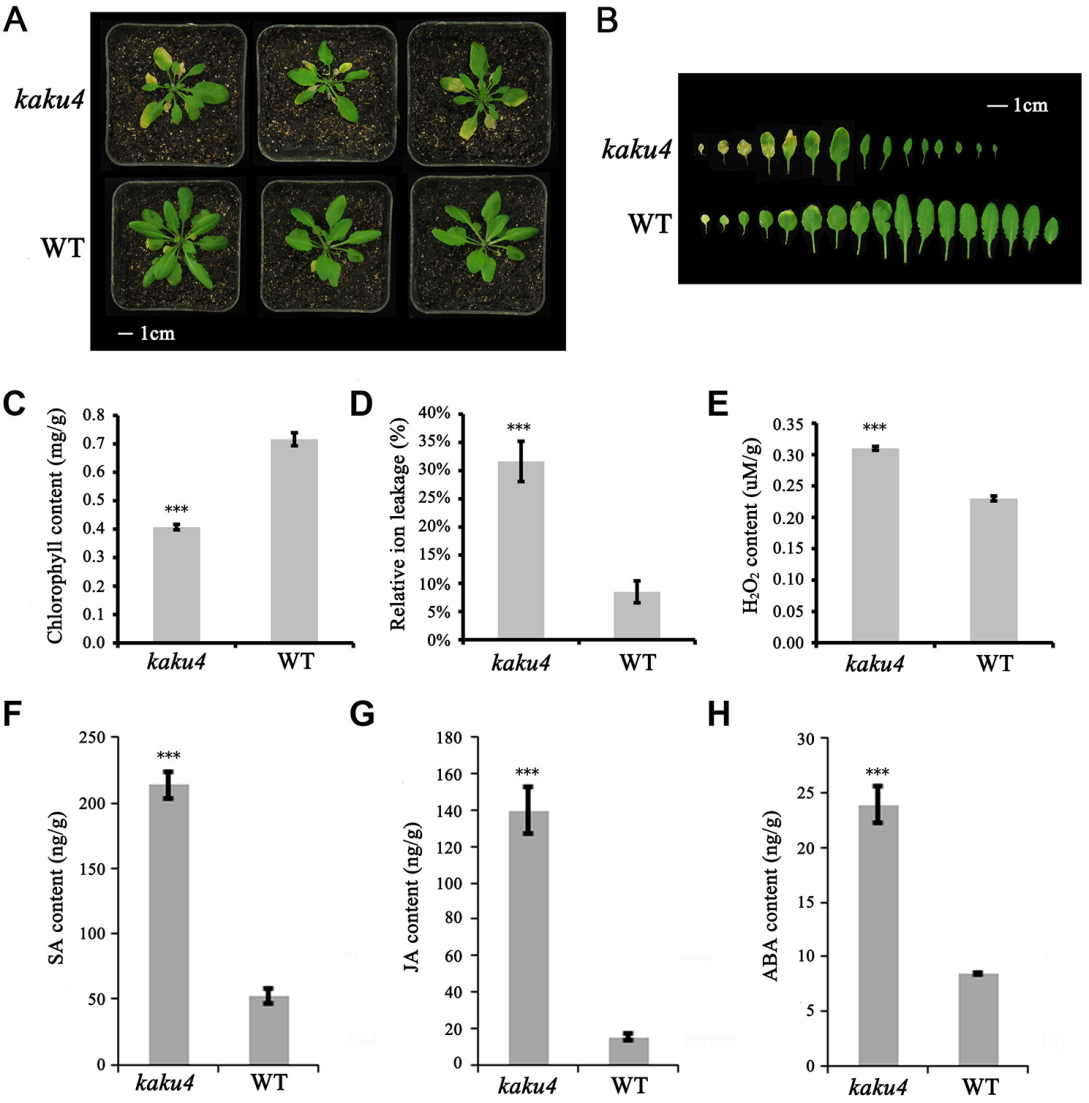
Phenotypic analysis of the *kaku4* mutant and WT in leaf senescence. (**A**) Age-dependent senescence phenotype of the sixth-week *kaku4* mutant and WT. The length of the white bar represents 1 cm. (**B**) Rosettes leaves of the sixth-week *kaku4* mutant and WT. Bar = 1 cm. (**C**) Chlorophyll content in the sixth-week rosette leaves of the *kaku4* mutant and WT. (**D**) Relative ion leakage in the sixth-week rosette leaves of the *kaku4* mutant and WT. (**E**) H_2_O_2_ content in the sixth-week rosettes leaves of the *kaku4* mutant and WT. (**F**-**H**) Content of the plant hormone: SA (**F**), JA (**G**), and ABA (**H**) in the sixth-week rosettes leaves of *kaku4* mutant and WT. Significant difference between the *kaku4* mutant and WT were determined according to Student’s t-test. *P-value ≤ 0.05, **P-value ≤ 0.01, ***P-value ≤ 0.001. The pair-wise ANOVA test for the genotypic variation between *kaku4* mutant and WT (**C**-**H**) were showed in Supplementary Table S11

To investigate the possible role of the *KAKU4* gene in leaf development, we performed a genetic analysis of the age-triggered leaf-senescence phenotype for the SALK T-DNA insertion line *kaku4* mutant (SALK_076754) (Supplementary Figure S1A) and wide-type (WT) plants. Compared to WT, *kaku4* mutant plants exhibited a significantly accelerated leaf-senescence phenotype. In 4-week-old plants, the cotyledons of the *kaku4* mutant had begun to turn yellow (Supplementary Figure S1D). In the sixth week, some rosette leaves of *kaku4* mutant turned yellow, while most leaves of WT plants remained green (Fig. 1A and B, Supplementary Figure S1E). Additionally, whole plant and leaves of the *kaku4* mutant were smaller than those of WT plants (Fig. 1A and B). We also examined some senescence-related parameters to validate the early-senescence phenotype of the *kaku4* mutant. Consistent with the accelerated leaf-senescence phenotype, the chlorophyll content in rosette leaves of the *kaku4* mutant was significantly lower than that in the WT (Fig. 1C), and membrane-ion leakage was higher in the *kaku4* mutant than in the WT (Fig. 1D).

To study the possible signaling pathways of KAKU4-mediated leaf senescence, we measured several aspects related to leaf senescence, such as H_2_O_2_ and hormones. H_2_O_2_ is an important regulator of leaf senescence [32]. Many aging-triggered leaf senescence processes are mediated by the H_2_O_2_-signaling pathway [33, 34]. Using a quantitative assay, we assessed H_2_O_2_ content in sixth-week rosette leaves of the *kaku4* mutant and WT plants, and found that the *kaku4* mutant produced much more H_2_O_2_ than the WT (Fig. 1E). The measurement result for H_2_O_2_ indicated that knockdown of *KAKU4* gene could accelerate leaf senescence by inducing H_2_O_2_ levels. Phytohormones also function as important regulators in leaf senescence, and SA, ABA, and JA accelerate leaf senescence [33, 35, 36]. Thus, we measured the levels of several hormones in the sixth-week rosette leaves of the *kaku4* mutant and WT plants (Fig. 1F-H). The contents of SA, JA, and ABA in *kaku4* mutant were 4-fold, 9-fold, and 3-fold higher than those in WT, respectively. In the meanwhile, we used one-way ANOVA with Bonferroni correction for the statistical analysis, the results showed that the difference of the phenotypes between *kaku4* mutant and WT were significant (Supplemental Table S11).

In addition, we applied a transgenic strategy to complement the *kaku4* mutant phenotype. We generated several *KAKU4*-complemented lines (*35S::KAKU4/kaku4-1* and *35S::KAKU4/kaku4-2*) and *KAKU4*-overexpression lines (*35S::KAKU4/WT-1* and *35S::KAKU4/WT-2*) under the control of the 35S promoter (Supplementary Figure S1B) against the background of the *kaku4* mutant and WT, respectively. The gene expression levels of both types of transformation lines together with the *kaku4* mutant and WT were validated with qRT-PCR (Supplementary Figure S1C). The results of phenotypic analysis showed that the accelerated leaf-senescence phenotype was rescued in *KAKU4*-complemented lines, and chlorophyll content was higher than that of the *kaku4* mutant. In the meantime, *KAKU4-*overexpression lines exhibited a slightly delayed leaf-senescence phenotype with relatively higher chlorophyll content than that in WT plants (Supplementary Figure S1E and F).

To further confirm that the mutation of *KAKU4* accelerates leaf senescence, we employed CRISPR/Cas9 gene editing approach to knock down the *KAKU4* gene and generated specific and heritable targeted mutations in Arabidopsis. We designed one editing site and successfully obtained three CRISPR/Cas9 mutants, including *kaku4-03*^Cas9^, *kaku4-04*^Cas9^, and *kaku4-05*^Cas9^ (Supplementary Figure S2A). Compared to WT, *kaku4* CRISPR/Cas9 mutants showed accelerated leaf-senescence phenotype as well as the T-DNA insertion line, no matter for the cotyledons of *kaku4* mutants in the young plants (Supplementary Figure S2B) or for the rosette leaves of *kaku4* mutants in the mature plants (Supplementary Figure S2C and S2D). In the meanwhile, we examined the chlorophyll content in rosette leaves of both T-DNA insertion line and CRISPR/Cas9 mutants, and the result validated the early-senescence phenotype of the *kaku4* mutants (Supplementary Figure S2E).

Overall, our results demonstrated the importance of KAKU4 as a potential negative regulator in age-triggered leaf senescence in Arabidopsis.

### KAKU4 mediates some key biological processes

**Fig. 2:**
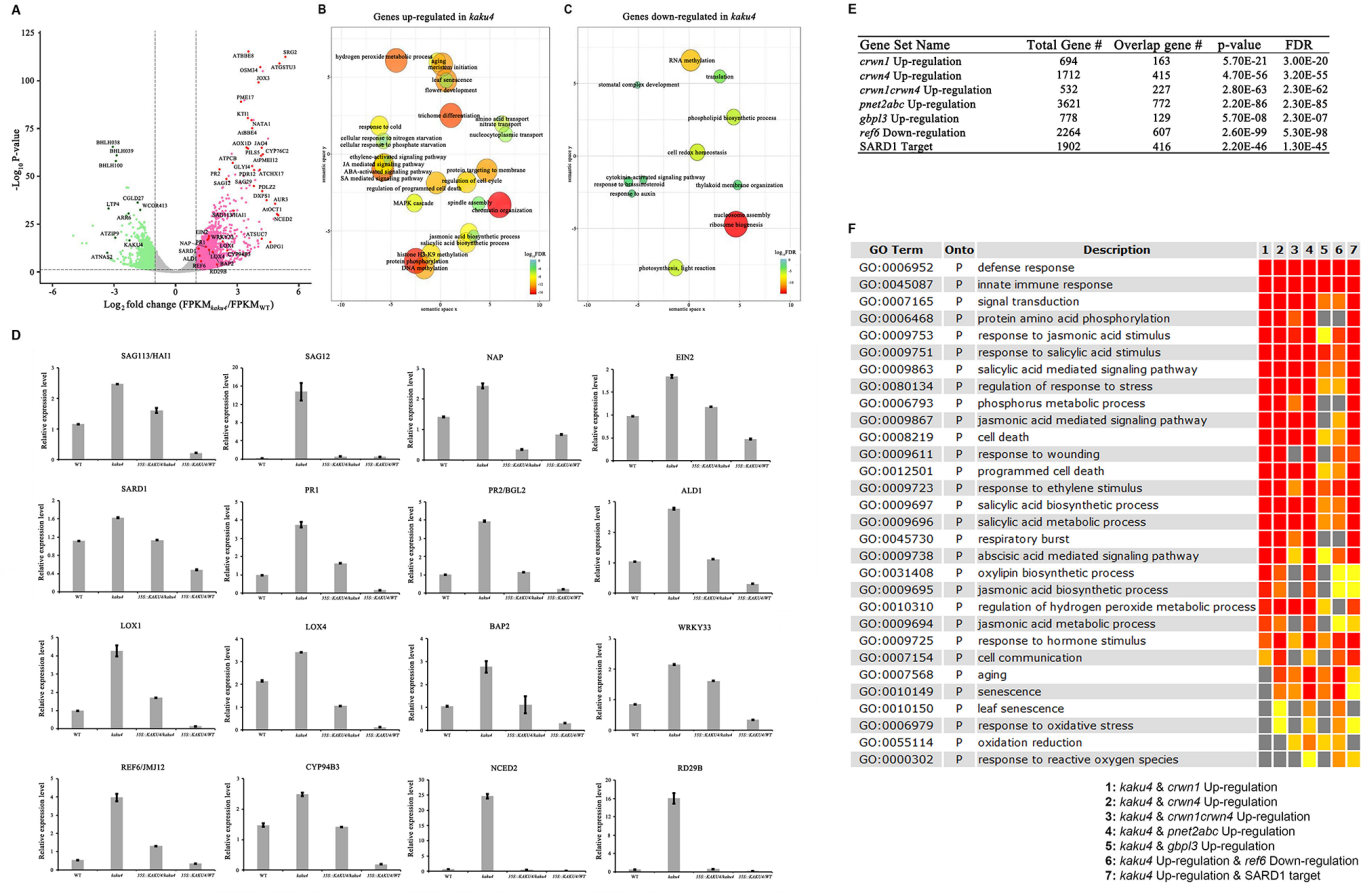
RNA-seq based transcriptome data analysis of the *kaku4* mutant and WT. (**A**) mRNA-seq analyses of gene expression in the *kaku4* mutant using stringent cutoff criteria (|log_2_(fold change)| ≥ 1 and P-value ≤ 0.05). Dashed lines indicate a log_2_(fold change) of ± 1. Red dots indicate the 3281 upregulated genes in the *kaku4* mutant. The green dots indicate the 2073 downregulated genes in the *kaku4* mutant. (**B**) and (**C**) GO enrichment analyses for genes that were upregulated (**B**) and downregulated (**C**) in the *kaku4* mutant by agriGOv2 and REVIGO. The scatter plot shows the cluster representatives in two-dimensional space derived by applying multidimensional scaling to a matrix of the significant GO terms with semantic similarities. The color and size of the bubble indicate the log_10_FDR (legend in bottom right-hand corner). The colors from red to green represent the significance level of the GO terms from high to low. Fisher’s exact test was conducted, and the P-value was adjusted using the Benjamini-Yekutieli method, FDR ≤ 0.05. (**D**) Real-time RT-PCR for selected genes among WT, *kaku4* mutant, *KAKU4*-complementation, and *KAKU4*-overexpression lines; the error bars represent the standard deviations of three replicates. The transcripts are as follows: *SAG113/HAI1* - AT5G59220; *SAG12* - AT5G45890; *NAP* - AT1G69490; *EIN2* - AT5G03280; *SARD1* - AT1G73805; *PR1* - AT2G14610; *PR2/BGL2* - AT3G57260; *ALD1* - AT2G13810; *LOX1* - AT1G55020; *LOX4* - AT1G72520; *BAP2* - AT2G45760; *WRKY33* - AT2G38470; *REF6/JMJ12* - AT3G48430; *CYP94B3* - AT3G48520; *NCED2* - AT4G18350; and *RD29B* - AT5G52300. The primers for each gene are listed in Supplementary Table S4. (**E**) Comparative analysis of the differential expressed genes (DEGs) of *kaku4* mutant and *crwn1*, *crwn4*, *crwn1crwn4*, *pnet2abc*, *gbpl3*, and *ref6* mutants, and comparative analysis of the upregulated DEGs of *kaku4* mutant and the target genes of SARD1. Significance level of the overlap using the custom SEA tool in the plantGSAD database. (**F**) SEA compare analysis of GESA enrichment analysis results for the upregulated DEGs in *kaku4* mutant, the overlap DEGs in *kaku4* mutant and *crwn1*, *crwn4*, *crwn1crwn4*, *pnet2abc*, *gbpl3*, and *ref6* mutants, and overlapping genes between upregulated DEGs in *kaku4* mutant and SARD1 target genes

Transcriptomic data analysis was conducted to explore the possible downstream genes regulated by KAKU4 during leaf senescence. Rosette leaves of the sixth-week *kaku4* mutant and WT plants were sampled for RNA-seq with three independent biological replicates for each sample (Supplementary Table S1). We performed differential expression analyses on RNA-seq data using DESeq2 [37] with the cutoff: |log_2_(fold change)| ≥ 1 and P-value ≤ 0.05. Compared to the WT, 3,281 genes were upregulated and 2,073 genes were downregulated in *kaku4* mutant (Supplementary Table S2). Among these differentially expressed genes, we found that some iron homeostasis related BHLH genes such as *BHLH038*, *BHLH039*, and *BHLH100* [38] were significantly downregulated with large fold changes (log_2_(fold change) ≤ -3) (Fig. 2A). Likewise, some upregulated genes showing the large fold changes (log_2_(fold change) ≥ 3), are mainly related to leaf senescence, programmed cell death (PCD), and plant hormone pathways, including *SRG2*, *AtGSTU3*, *KTI1*, *JOX3*, *JAO4*, *NATA1*, and *NCED2*. The *SRG2* gene encodes a protein similar to beta-glucosidase that is expressed in the senescing organs of *Arabidopsis* plants [39]. We found that *SRG2* was significantly upregulated in the 6-week-old rosette leaves of the *kaku4* mutant, as well as the senescence-associated reference gene *AtGSTU3*, which participates in PCD and the toxin catabolic process [40]. In addition, the genes with large fold change between the WT and *kaku4* mutant also included some hormone biosynthesis and signaling pathways related genes, such as the ABA synthesis gene *NCED2*, SA-related genes (*KTI1*), and JA-related genes (*NATA1*, *JOX3*, and *JAO4*) [41–44]. This result indicated that the mutation of *KAKU4* significantly affected the expression of senescence- and hormone-related genes. We further performed Gene Ontology (GO) enrichment analysis for differentially expressed genes (DEGs) to infer related biological processes in KAKU4-mediated leaf senescence (Fig. 2B and C). For genes that are upregulated in *kaku4* mutant, the enriched GO terms were mainly related to chromatin organization and histone modification, phytohormones (SA, JA, ABA, ethylene, and so forth), programmed cell death, hydrogen peroxide metabolic process, leaf senescence, and so forth (Fig. 2B, Supplementary Table S3). The results of GO enrichment analysis suggested that phytohormone signaling, H_2_O_2_-mediated signaling, and leaf senescence were activated in the *kaku4* mutant, which was coincident with the results of phenotype analysis between the *kaku4* mutant and WT (Fig. 1). Additionally, many other biological processes were also activated in the *kaku4* mutant, and the related GO terms include nitrate transport, cellular response to nitrogen starvation, cellular response to phosphate starvation, regulation of cell cycle, and regulation of programmed cell death (Fig. 2B, Supplementary Table S3). Meanwhile, the genes downregulated in the *kaku4* mutant were mainly associated with ribosome biogenesis, RNA methylation, “photosynthesis, light reaction”, phospholipid biosynthetic process, and cytokinin-activated signaling pathway (Fig. 2C, Supplementary Table S3). Taken together, genome-wide transcriptome analysis revealed that KAKU4 acts as a regulator for many biological processes including chromatin remodeling, hormone signaling, and leaf senescence.

We further used additional biological replicate samples and carried out qRT-PCR analyses for some selected marker genes (Supplementary Table S4). We added the leaf samples from the mutant complementation and overexpression lines. As shown in Fig. 2D and 16 genes were upregulated in the *kaku4* mutant, while the mRNA levels for most of them were rescued nearly to the WT level in the complementation lines, and much lower levels in the overexpression lines. The expression levels of most selected genes validated the RNA-seq results very well. Besides several key senescence marker genes such as *SAG113/HAI1* and *SAG12*, some hormone pathways and cell death related genes were also validated. For example, *SARD1*, *PR1*, *PR2*, and *BAP2* were both involved in the SA pathway and in regulating plant immunity [45–47]. *CYP94B3*, *LOX1*, and *LOX4* were involved in JA synthesis, and have been reported to be related to leaf senescence [48–50]. In addition, ABA biosynthesis (*NCED2*) and signal pathway genes (*RD29B*, *SAG113/HAI1*) were included, as was the ethylene signal transduction gene (*EIN2*) [44, 51–53].

The results of transcriptome analysis matched our phenotypes well, and the mutation of *KAKU4* gene resulted in upregulation of multiple genes, possibly promoting leaf senescence.

### Functional crosstalks between KAKU4 and CRWNs/PNET2/GBPL3/REF6/SARD1 through comparing of the transcriptome data

KAKU4 protein interacts with lamin-like proteins CRWNs, PNET2_A/B, and nuclear basket protein GBPL3, and these proteins are required to balance plant growth and stress responses [16, 18, 21]. In order to investigate whether there are some functional crosstalks between KAKU4 and CRWNs, PNET2, GBPL3, we compared the transcriptome data related to *kaku4* mutant and *crwn1*, *crwn4*, *crwn1crwn4*, *pnet2abc*, *gbpl3* mutants (Supplementary Table S6). We reanalyzed the transcriptome data of *crwn1*, *crwn4*, and *crwn1crwn4* mutants [21], and obtained the DEGs of *crwn* mutants (*crwn1*, *crwn4*, *crwn1crwn4*). The DEGs of the *pnet2abc* and *gbpl3* mutants were obtained from literatures [16, 18]. Between the *kaku4* mutant and the mutants of these associated genes, we found that there were significant overlaps of their upregulated DEGs (Fig. 2E).

In the meanwhile, we considered the fact that SARD1 is a key regulator of the expression of SID2 and can promote SA synthesis [54]. We obtained a list of the target genes of SARD1 from literature [55] and found that there existed significant overlap between the upregulated DEGs of *kaku4* mutant and the target genes of SARD1 (Fig. 2E). Furthermore, the loss of function of H3K27me3 demethylase REF6 increased H3K27me3 levels at all of the target senescence associated genes (*SAGs*) [56]. REF6 promotes leaf senescence by directly activating major senescence regulatory and functional genes in Arabidopsis. We carried out real-time qRT-PCR analyses for the *JMJ12/REF6* gene, and the result showed that *JMJ12/REF6* gene was upregulated in the *kaku4* mutant, while in the KAKU4 complementation lines, the mRNA level of the *JMJ12/REF6* gene was partially rescued, and the *JMJ12/REF6* gene showed a much lower level of mRNA in the *KAKU4* overexpression lines (Fig. 2D). We obtained the transcription profiles of the mutant of *JMJ12/REF6* from literature [56]. The significant overlap was found between the upregulated DEGs in *kaku4* mutant and downregulated DEGs in *ref6* mutant (Fig. 2E).

We further applied the SEA compare tool in the PlantGSAD database [57] to study the biological processes of the overlap gene between these genes and upregulated DEGs in *kaku4* mutant. We found that these overlapping genes enriched biological process terms such as “defense response”, “immune response”, “cell death” and “regulation of hydrogen peroxide metabolic process”. We also found many biological process terms related to hormones such as “salicylic acid mediated signaling pathway”, “jasmonic acid mediated signaling pathway”, “response to ethylene stimulus”, “response to abscisic acid stimulus”, etc. (Fig. 2F).

Overall, we compared the multiple transcriptome datasets and the results indicate that there were some functional crosstalks between KAKU4 and its associated proteins (CRWN1/4, PNET2, GBPL3, SARD1, REF6), in some important biological processes such as defense response, cell death, and response to SA, JA, ABA, and forth.

### Distinct H3K27me3 profiles in the *kaku4* mutant and WT plants

**Fig. 3:**
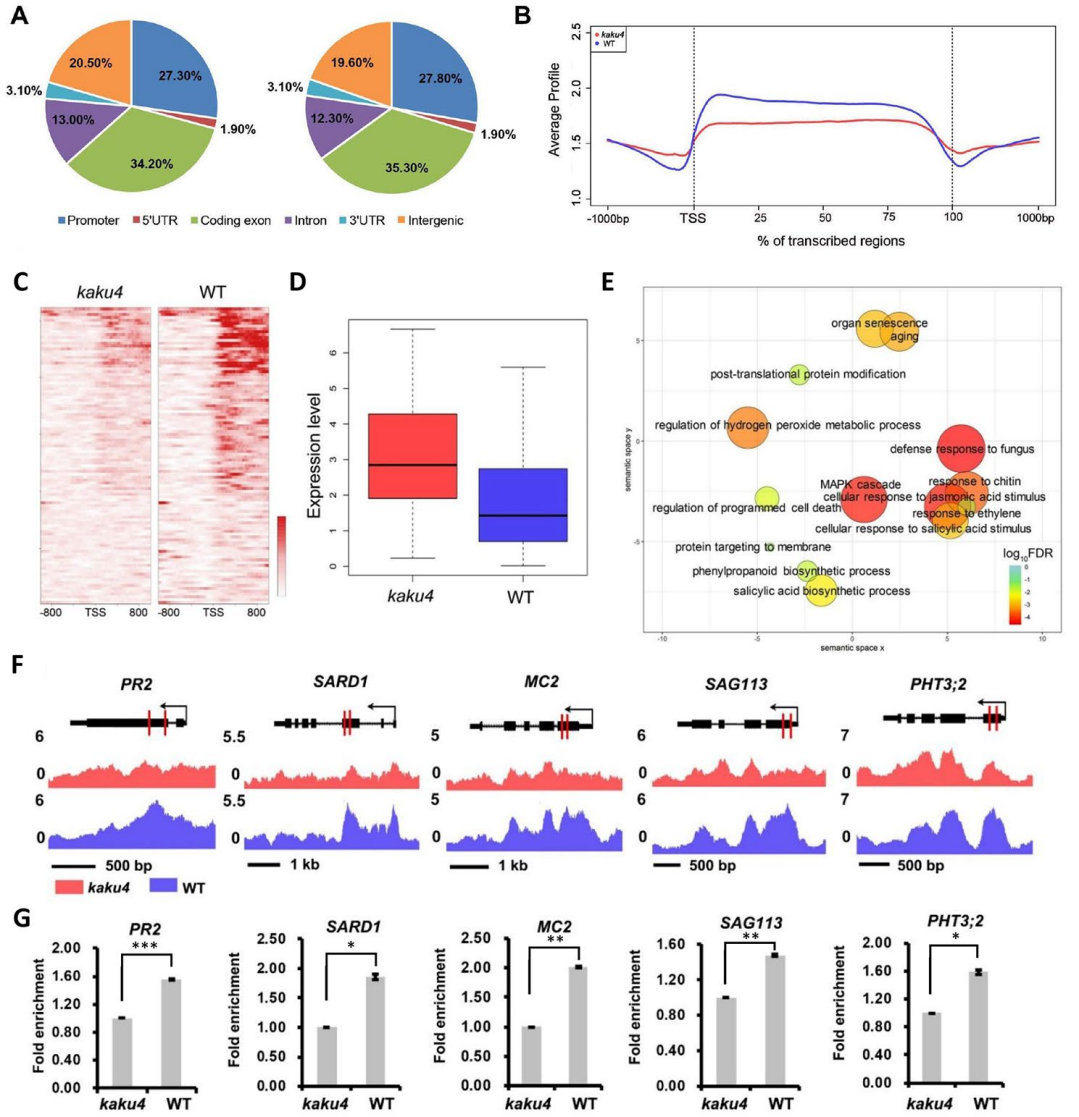
ChIP-seq analysis of H3K27me3 in the *kaku4* mutant and WT. (**A**) The distribution of H3K27me3 within different regions (Promoter, 5’UTR, Coding exon, Intron, 3’UTR and Intergenic) of *Arabidopsis* genome in the *kaku4* mutant and WT. (**B**) Distribution of H3K27me3 among the *Arabidopsis* genes. A meta-gene profile was generated using the normalized sequencing density of H3K27me3 in the *kaku4* mutant and WT. The gene body was converted into a percentage to standardize genes of different lengths. The 1 kb upstream and downstream regions of the gene are included. (**C**) The heatmaps display H3K27me3 signal around TSSs in the *kaku4* mutant and WT for genes upregulated and with decreased H3K27me3 accumulation in the *kaku4* mutant. For each gene, the H3K27me3 signals are displayed along − 1 kb to 1 kb regions around the TSSs. (**D**) The gene expression values in the *kaku4* mutant and WT are shown for genes upregulated and with decreased H3K27me3 accumulation in the *kaku4* mutant. (**E**) GO enrichment analyses for genes upregulated and with decreased H3K27me3 accumulation in the *kaku4* mutant by agriGO and REVIGO. The scatterplot shows the cluster representatives in a two-dimensional space derived by applying multidimensional scaling to a matrix of significant GO terms with semantic similarities. Bubble color and size indicates the log_10_(FDR) (legend in bottom right). (**F**) Genes with decreased H3K27me3 accumulation in the *kaku4* mutant are shown in the UCSC genome browser. The locations of ChIP–qPCR primers for H3K27me3 validation are marked in red lines. (**G**) ChIP–qPCR validation of H3K27me3 deposition for genes with decreased H3K27me3 in *kaku4* mutant. Significant difference between the *kaku4* mutant and WT were determined according to Student’s t-test. *P-value ≤ 0.05, **P-value ≤ 0.01, ***P-value ≤ 0.001

KAKU4 can physically interact with CRWN1 and CRWN4 [14], which are in the same complex with a PWWP-containing protein PWO1 [26]. PWO1 interacts with several subunits of PRC2 (i.e., CLF, SWN, and MEA) [27]. We performed immunoblotting with specific antibody to detect trimethylate lysine 27 on histone H3 (H3K27me3) in the leaves of *kaku4* mutant and WT. As shown in Supplementary Figure S3, the H3K27me3 level in *kaku4* mutant was significantly lower than that in WT. We further performed H3K27me3 ChIP-seq using the sixth-week rosette leaves of the *kaku4* mutant and the WT. A total of 3,748 and 4,695 enriched regions of H3K27me3 were identified in the *kaku4* mutant and the WT, which were associated with 4,560 and 5,797 genes, respectively (Supplementary Table S7 and S8). More H3K27me3 deposited regions were found in WT than in the mutant. Further, we characterized the H3K27me3 distribution in different functional regions. The *Arabidopsis* genome was categorized into six genomic subregions: promoter, 5’ untranslated region (5’UTR), 3’UTR, exon, intron, and intergenic region, based on the physical positions of the genes. The distribution of H3K27me3 was similar in the *kaku4* mutant and the WT, mainly distributed in the exon region, with the exception of slight differences in the coding exon, intergenic and other regions (Fig. 3A).

We performed metagene analyses to compare the level of normalized H3K27me3 signal along the genetic region. It was found that H3K27me3 reached a higher level in WT than in the *kaku4* mutant in the gene body, but the deposition upstream of the TSS was slightly lower than that in the mutant (Fig. 3B). Then, we identified genes with these differential H3K27me3 regions located within 2 kb upstream of TSS and the gene bodies as differential H3K27me3-deposited genes. A total of 820 genes with higher H3K27me3 deposition were identified in the WT and 16 genes in the *kaku4* mutant (Supplementary Table S9). In the *kaku4* mutant, significantly more genes were associated with decreased H3K27me3 deposition than with increased deposition. The presence of H3K27me3 is usually correlated with gene silencing in animals and plants [58].

We conducted combination analyses of transcriptomic and epigenomic data. We compared H3K27me3-changed genes and differentially expressed genes between the *kaku4* mutant and the WT, and identified 104 genes with decreased H3K27me3 deposition (Fig. 3C) and higher expression levels in the mutant (Fig. 3D and Supplementary Table S10). Their upregulated expression might be affected by KAKU4-involved H3K27me3 deposition. GO enrichment showed that GO terms associated with SA response, SA biosynthesis, JA response, defense response, aging, and organ senescence were enriched (Fig. 3E). Some processes closely related to aging, such as H_2_O_2_ metabolism and programmed cell death, were also significantly enriched (Fig. 3E). In addition, many genes related to ABA metabolism and signal transduction pathway were identified with lower H3K27me3 deposition and were upregulated in the *kaku4* mutant, including the ABA synthesis gene *NCED2*, the ABA 8’-hydroxylase gene *CYP707A2*, and ABA signal pathway genes such as *SAG113/HAI1, HAI2*, and *RD29B*.

We selected several genes (*PR2*, *SARD1*, *MC2*, *SAG113*, and *PHT3;2*) for ChIP-qPCR validation. *PR2* is the marker gene activated in response to pathogens. *SARD1* is a key regulator of the expression of *SID2* and can promote SA synthesis [54]. *PHT3;2* encodes a mitochondrial phosphate transporter that is highly expressed in senescent leaves [59]. *SAG113*/*HAI1* functions as a negative regulator of osmotic stress and ABA signaling. The metacaspases MC2 and MC1 antagonistically control programmed cell death in *Arabidopsis* [60]. The ChIP-qPCR results of the selected genes showed a decrease in H3K27me3 in the *kaku4* mutant than in the WT, which confirmed the ChIP-seq results (Fig. 3F and G).

In summary, these results indicated that KAKU4 might regulate SA signaling, JA signaling, ABA signaling, and senescence by affecting H3K27me3 deposition.

## Discussion

In this study, we found that the mutation of *KAKU4* gene resulted in an accelerated leaf senescence phenotype, with higher levels of H_2_O_2_ and some hormone contents. RNA-seq analysis showed that multiple genes were differentially expressed between the *kaku4* mutant and the WT plants in 6-week-old rosette leaves. In particular, the upregulated genes in the mutant were significantly enriched in some hormone pathways (SA, JA, ABA, and ethylene), H_2_O_2_ metabolism, programmed cell death, leaf senescence, and flowering, among others. In addition, we also concerned that knockdown of *KAKU4* may affect the biological processes regulated by other lamin-like proteins such as CRWNs, PNET2, and nuclear basket protein GBPL3, etc. We conducted data-mining analysis and the results showed that there are some functional crosstalks between KAKU4 and its associated proteins (CRWN1/4, PNET2, GBPL3), including defense response, cell death, and response to SA and JA, etc. Further ChIP-seq and ChIP-qPCR analyses showed that KAKU4 was involved in H3K27me3 deposition in a series of genes associated with hormone pathways, H_2_O_2_ metabolism, leaf senescence, and so on.

KAKU4 physically interacts with two lamin-like proteins: CRWN1 and CRWN4 [14]. CRWN1 is reported to be involved in SA-mediated plant immune responses [61]. CRWN1 interacts with NTL9 and SNI1, forming a complex to repress the expression of *PR1*. In addition, it inhibits the function of NPR1, which promotes *PR1* expression mediated by TGA1. CRWNs may also participate in the regulation of SA biosynthesis [21]. In *Arabidopsis*, the double-mutants *crwn1crwn3*, *crwn2crwn3*, and *crwn2crwn4* exhibit accelerated cell death and increased ROS accumulation [23]. In particular, CRWNs were reported to specifically localize and build a meshwork structure at the nuclear periphery, regulating chromatin distribution and gene expression through fluorescence in situ hybridization and RNA-seq analyses [25]. In addition, CRWNs physically interact with a PWWP domain containing protein PWO1 [26]. Through the mediation of PWO1, CRWN1 and CRWN4 may be associated with PRC2 and H3K27me3 accumulation. The expression of a substantial number of H3K27me3 targets is affected by either loss of PWO1 or CRWN1/CRWN2 [26]. The H3K27me3 accumulation levels near the genes involved in SA biosynthesis are decreased in *crwn* mutants [29]. Our epigenomic data analysis results presented here showed that knockdown of *KAKU4* significantly modulated the H3K27me3 modification in Arabidopsis genome. The combination analyses of epigenomic and transcriptomic data showed that, the genes with higher H3K27me3 deposition in WT and up-regulated in *kaku4* mutant, were significantly associated with some hormone pathways (such as SA signaling and biosynthesis pathways), program cell death and leaf senescence, etc. A key SA biosynthesis regulator, SARD1, shows decreased H3K27me3 accumulation in *crwn1crwn2* [29], while the expression of SARD1 increases markedly in *crwn1crwn2* [21]. Interestingly, we found that *SARD1* was upregulated in the *kaku4* mutant with lower deposition of H3K27me3, as well as many genes related to defense and cell death. These results indicated that KAKU4 plays a key role in regulating senescence-associated hormone pathways in the leaves with the mediation of H3K27me3 deposition.

**Fig. 4:**
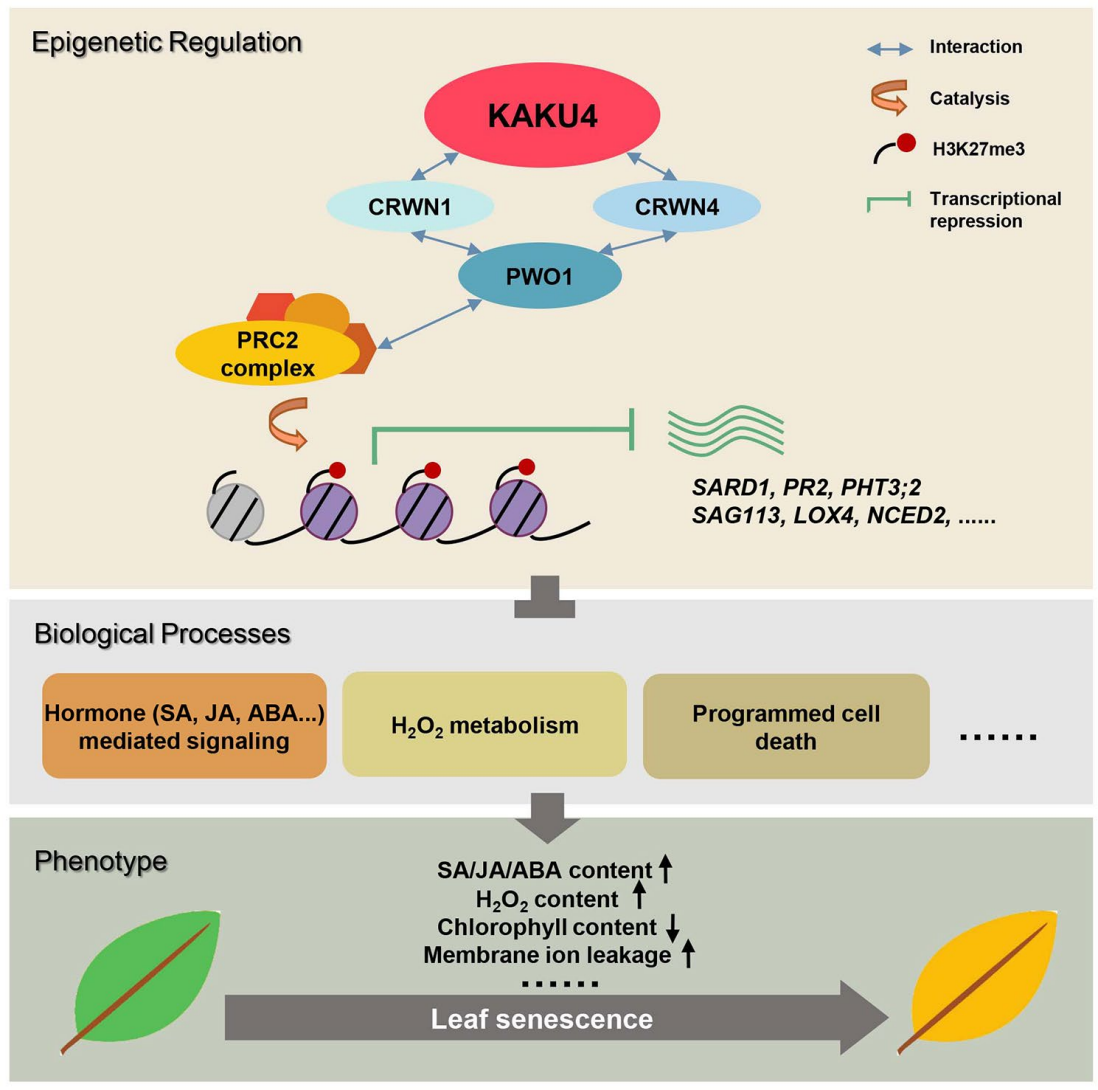
Model for the role of KAKU4 during leaf senescence. KAKU4, CRWN1, CRWN4, PWO1, and PRC2 might form complexes through protein-protein interaction that promote H3K27me3 deposition on a series of genes, and inhibit the expression of these genes. These genes were involved in the regulation of some biological processes, such as hormone-mediated signaling pathways (SA, JA, ABA, and so forth), PCD, and H_2_O_2_ metabolism, which suppress the production of related hormones and H_2_O_2_, while maintaining chlorophyll content to ultimately prevent the senescence of plant leaves

Taken together, we proposed a model for the role of KAKU4 in leaf senescence (Fig. 4). KAKU4 interacts with CRWN1 and CRWN4, which physically interact with PWO1, possibly promoting the deposition of H3K27me3 on a series of genes. This in turn inhibits the expression of some key genes (such as *SARD1*, *PR2*, *LOX4*, *NCED2*, *SAG113*/*HAI1*, *PHT3;2*, and others), leading to the inhibition of some biological processes, such as hormone-signaling pathways, PCD, and leaf senescence. The knockdown of the *KAKU4* gene causes a series of phenotypic changes, such as SA, JA, ABA and H_2_O_2_ accumulation, increased ion leakage at the membranes, and reduced chlorophyll content, resulting in leaf senescence. Our findings expand the knowledge for the role of KAKU4 in regulating key genes related to hormone pathways, PCD, and leaf senescence.

## Materials and methods

### Plant materials and growth conditions

Surface-sterilized *Arabidopsis thaliana* (Col-0, *kaku4* mutant lines, *KAKU4*-complementation lines, and *KAKU4*-overexpression lines) seeds were placed on half-strength MS (Murashige and Skoog) medium (pH 5.7 to 5.8, 1% [w/v] sucrose, and 0.7% agar). Following a three-day stratification period at 4 °C, the seeds were transferred to a conditioning chamber that had a 16-hour light (22 °C)/8-hour dark (19 °C) cycle. The seedlings were planted in soil after germination for ten days.

### Identification of the *kaku4* T-DNA insertion mutant

From the Arabidopsis Biological Resource Center (ABRC), the *kaku4* (SALK_076754) seeds were acquired. Using two sequential PCR experiments, homozygous T-DNA insertion mutant plants were verified. Two gene-specific primers were used in one assay: RP (SALK_076754-RP) and LP (SALK_076754-LP). One gene-specific primer (SALK076754-RP) and one T-DNA-specific primer (LB) were utilized in the other test. Supplementary Table S4 contains a list of all primers utilized in this investigation.

### Construction of transgenic *KAKU4-*complementation lines and *KAKU4*-overexpression lines

Amplification of the cDNA region of KAKU4 was achieved by PCR using specific primers (Supplementary Table S4) to produce the genetic complementation and overexpression lines of the protein. For the control of the 35 S promoter, the acquired DNA sequence was ligated into the *XbaI* and *KpnI* sites of the super-1300 vector (pCambia1300). After the DNA construct was confirmed through sequencing, it was electroporated into *Agrobacterium tumefaciens* GV3101, which underwent floral infiltration to produce the *KAKU4*-complementation lines and *KAKU4*-overexpression lines [62]. Hygromycin resistance was used to screen the transformed plants, and PCR was used to confirm the results. For further investigation, homozygous T2 seeds of transgenic plants were utilized.

### Construction and identification of the *kaku4* CRISPR/Cas9 mutant

The coding sequences of *KAKU4* were chosen as the target and cloned into the plasmid (pCBSG12B) to generate *kaku4* mutant by the clustered regularly interspaced short palindromic repeats (CRISPR)/associated protein9 (Cas9) gene editing approach. The *kaku4-03*^Cas9^, *kaku4-04*^Cas9^, and *kaku4-05*^Cas9^ were generated in Columbia background and confirmed by PCR assay, using the gene-specific primers: ka-ex1_cas_F2 and ka-ex1_cas_R2 (listed in Supplementary Table S4).

### Chlorophyll content, ion leakage, H_2_O_2_ content measurement

The 6-week-old rosette leaves were homogenized in liquid nitrogen and then incubated in 80% acetone (v/v) for an entire night at 4 °C in order to determine the chlorophyll content. Next, centrifuge at 2700 g for 3 min. Following measurements of absorbance at 646 and 663 nm, the chlorophyll concentration was computed as follows: Chlorophyll a (mg/mL) is equal to 12.21×A_663_ − 2.81×A_646_, while Chlorophyll b (mg/mL) is equal to 20.13×A_646_ − 5.03×A_663_. Chlorophyll a + Chlorophyll b equals the chlorophyll content (mg/mL).

Electrolytes that had leaked from leaves were measured in order to determine membrane ion leakage. The initial conductivity was measured after the third and fourth rosette leaves from the plants were submerged for three hours at 23 °C in three milliliters of 400 mM mannitol with light shaking. The total conductivity was measured following a 10-minute boil. The percentage of initial conductivity compared to total conductivity was used to calculate conductivity [63].

Using an Amples Red H_2_O_2_/peroxidase assay kit (Molecular Probes) and following the manufacturer’s instructions, the H_2_O_2_ production was quantified [64].

## Plant hormone content measurement

To determine the hormone content, 6-week-old rosette leaves from Arabidopsis plants cultivated in soil were harvested. After homogenizing the rosette leaves in liquid nitrogen, 500 µl of the extract solution and 50 µl of the internal standard working solution were added. The mixture was vortexed and shaken for 30 min at 4 °C. 1 ml of the extract CHCl_3_ was then added, and the mixture was vortexed and shaken for 30 min at 4 °C again. Gather the bottom layer of liquid after centrifugation, then use helium to dry it at room temperature. Following their dissolution in 0.1 ml of MeOH, the samples were filtered through a 0.1 µM filter.

An UPLC-HRMS system with a heated electrospray ionization (HESI) source (UPLC, Waters ACQUITY UPLC i-Class, Milford, MA, USA; MS, Thermo Fisher Q-Exactive, Bremen, Germany) was used to analyze the samples. Chromatographic separation with a Poroshell 120 EC-C18 column (3.0 × 75 mm, 2.7 μm, Agilent, USA) at a flow rate of 0.4 ml min^− 1^. For the analysis of SA, ABA, and JA, the mobile phases were 0.05% AA in water (phase A) and 0.05% AA in ACN (phase B). Use 0.1% FA in MeOH (phase B) and 0.1% FA in water (phase A) for ZT and OPDA detection. The gradient program was configured to start at 90% A at 0 min, go from 60% A at 6.25 min, then 10% A at 7.5 min, hold for 3 min, and back to the starting condition. The temperature in the column was 35 °C.

In MS analysis, both positive and negative ion modes were employed. The spray voltage (+) at 3.5 kV, spray voltage (-) at 3 kV, aux gas flow rate at 10 units, sheath gas flow rate at 30 arbitrary units, sweep gas flow rate at 5 units, gas heater temperature at 350 °C, capillary temperature at 320 °C, and S-lens RF level at 55% were the parameters set for the HESI source. For data acquisition, two scans were used: the full MS scan (resolution: 70,000, AGC target: 3E6, maximum IT: 100 ms, scan range: 50–750 m/z) and the targeted MS2 scan (resolution: 17,500, AGC target: 2E5, isolation window: 2.0 m/z).

## RNA extraction and RNA-seq analysis

For the purpose of creating independent biological samples, 150 mg of the 6-week-old Arabidopsis plants’ rosette leaves (WT or *kaku4* mutant) were collected and frozen in liquid nitrogen. Using the TRIZOL® reagent (Invitrogen, CA, USA) and Qiagen RNeasy columns (Qiagen, Hilden, Germany), total RNA was isolated and purified in accordance with the manufacturer’s instructions.

The Beijing Genomics Institute constructed the sequencing libraries, and 150-bp pair-end reads were sequenced using an IlluminaHiSeq™ 2000. Three biological replicates of the RNA-seq data were produced. TopHat2 v2.0.9 software was used to align the paired-end clean reads of RNA-seq to the reference genome (TAIR10) [65] and Cufflinks were used to calculate FPKM values [66]. TopHat2 and Cufflinks were both run with default parameters. The cutoff: |log_2_FoldChange| ≥ 1 and P-value ≤ 0.05, were used to identify the differential expressed genes (DEGs) between WT and *kaku4* mutant (Supplementary Table S2).

### Chromatin immunoprecipitation and ChIP-seq analysis

With a few minor adjustments, a native chromatin immunoprecipitation (ChIP) was carried out essentially as former publications [67]. Approximately 20 g of the rosette leaves from the plants at week six were ground into a fine powder using liquid nitrogen and then resuspended in TBS (10 mM Tris, pH 7.5, 3 mM CaCl_2_, 2 mM MgCl_2_, 0.1 mM PMSF, 2/5 tab of complete mini (Roche Applied Science, Indianapolis) with 0.5% Tween 40). Subsequently, the nuclei were purified using a sucrose gradient and digested for five minutes at 37 °C using ten units of micrococcal nuclease (Sigma, St Louis). The nucleosome samples were centrifuged after being incubated for 4 h with 4% protein A Sepharose (GH healthcare Bio-Sciences AB, Uppsala) and pre-immune rabbit serum (1:100 dilution). Anti-trimethyl-histone H3 (Lys 27) antibodies (Millipore, 07-449) were incubated with the supernatant for an overnight period at 4 °C. The control experiments employed an identical volume of pre-immune rabbit serum, which functioned as a nonspecific binding control in every ChIP experiment. Following that, the samples were incubated for four hours at 4 °C with 25% protein A Sepharose. Using an elution buffer (20 mM Tris-HCl at pH 7.5, 5 mM EDTA, 50 mM NaCl, 1% SDS) the immune complexes were extracted from the washed beads following centrifugation of the pellet (bound) fractions. Following phenol/chloroform extraction, ethanol precipitation was used to extract immunoprecipitated DNA. In 100 µl of TE buffer (pH 8.0), the ChIPed DNA was once again suspended, and used for library construction by the Beijing Genomics Institute, then sequenced using the Illumina system Novaseq 6000 with a 150-bp read length.

Using BOWTIE2 software [68] with default parameters, all clean ChIP-seq reads were mapped to the Arabidopsis genome TAIR10. To identify peaks, the reads were organized using the MACS program (bandwidth, 300 bp; model fold, 10, 30; *P* = 1.00e-5) [69]. The UCSC genome browser was used to visualize the ChIP-seq data [70]. Using CEAS software, the distribution of peaks found in the ChIP-seq along the Arabidopsis genome were characterized [71]. Genes that overlapped the peaks, including the 2-kb upstream and gene body regions, were thought to carry the epigenetic marks. Supplementary Table S7 displayed the related peak numbers and gene numbers. Supplementary Table S9 included a list of all differential H3K27me3-related genes, which were utilized for further analysis.

### Gene ontology analysis

Using agriGOv2 [72] and REVIGO [73], GO enrichment analysis of RNA-seq and ChIP-seq data was carried out. The custom analysis in PlantGSEA platform [74]was used to perform the gene set enrichment analysis of KEGG, Caf, transcript factor target, and MapMan. P-values of less than 0.05 as the cutoff indicated that a term was significantly enriched.

### Quantitative real-time PCR

Utilizing the Moloney Murine Leukaemia Virus (M-MLV; Invitrogen), reverse transcription was carried out. After being heated to 70 °C for two minutes, 10 µl samples containing 2 µg of total RNA and 20 pmol of random hexamers (Invitrogen) were cooled on ice for an additional two minutes. Next, incorporate the reaction buffer and M-MLV into a 20 µl total volume that includes 20 pmol random hexamers, 500 µM dNTPs, 200 units of M-MLV, 50 mM Tris-HCl (pH 8.3), 75 mM KCl, 3 mM MgCl_2_ and 5 mM dithiothreitol. After that, the samples were incubated for 1.5 h at 37 °C [75]. The cDNA samples were diluted to 8 ng/µL for real-time RT-PCR analysis.

Using gene-specific primers (Supplementary Table S4) and an ECO Real-Time PCR system (Illumina) in accordance with the manufacturer’s instructions, real-time PCR experiments were carried out. SYBR Green was used to perform three biological repeats with 8 ng of cDNA. To standardize all of the data for the real-time RT-PCR experiments, each sample’s internal control was Arabidopsis 18 S rRNA (Supplementary Table S4). The quantitative variation between replicates was assessed using the relative quantitation method (ΔΔCT) to evaluate the gene-expression levels in wild-type, *kaku4* mutant, *KAKU4*-complementation and *KAKU4*-overexpression lines.

### ChIP-qPCR

The prepared DNA in ChIP was subjected to quantitative PCR analysis using an ECO Real-Time PCR system (Illumina). The ChIP-qRCR gene-specific primers were displayed in Supplementary Table S4. Three independent repetitions of the experiments were conducted. 25 S was utilized as a negative control to calculate the relative fold enrichment (RFE) of modified histone-associated sequences in the bound fractions. 2^−ΔΔCT^± standard deviation (SD) was used to compute RFE, where ΔΔCT is equal to ΔCT (positive control) - ΔCT (negative control).

### Immunoblots

After being ground in liquid nitrogen, harvested leaves from both the *kaku4* mutant and WT were extracted using 2×SDS-PAGE loading buffer (P1040; Solarbio) at 100 °C for 10 min. The mixture was centrifuged at 12,000 rpm for 10 min. Proteins that were extracted underwent separation on 10% SDS-PAGE gels before being transferred to PVDF membranes (Millipore, 0.22 μm). At room temperature (24 °C), membranes were blocked in blocking buffer (5% milk dissolved in 1×TBST (Tris Buffered Saline with Tween 20) for one hour. Then the membranes were incubated in blocking buffer with antibodies against H3 (BE3015; EASYBIO) and H3K27me3 (07-449; Millipore) for 1.5 h at room temperature. Membranes were incubated for one hour at room temperature with TBST after being washed five times for three minutes each. Membranes were incubated for 1 h at room temperature with diluted 1/2,000 Goat Anti-Mouse IgG, HRP Conjugated (CW0102S; CWBIO) and Anti-Rabbit VHH HRP (KTSM1322; AlpalifeBio) following five rounds of washing in TBST for three minutes each. Membranes were incubated in electrochemiluminescence (ECL) buffer (BE6706; EASYBIO) for one minute after being rinsed five times in TBST.

### Electronic supplementary material

Below is the link to the electronic supplementary material.

Supplementary Material 1

Supplementary Material 2

Supplementary Material 3

Supplementary Material 4

Supplementary Material 5

Supplementary Material 6

Supplementary Material 7

Supplementary Material 8

Supplementary Material 9

Supplementary Material 10

Supplementary Material 11

Supplementary Material 12

Supplementary Material 13

Supplementary Material 14

Supplementary Material 15

## Data Availability

The RNA-seq and ChIP-seq raw data were deposited in the NCBI Gene Expression Omnibus with the accession number GSE250299.
