# Supplementary material for: KAKU4 regulates leaf senescence through modulation of H3K27me3 deposition in the Arabidopsis genome
Source: BMC Plant Biol. 2024 Mar 7;24:177. doi: 10.1186/s12870-024-04860-9 (PMC10919013; doi:10.1186/s12870-024-04860-9)
Supplement: Supplementary file 1 — Supplementary Material 1 [file 12870_2024_4860_MOESM1_ESM.docx]

## Supplementary Figure Legends

**Supplementary Figure S1. Phenotypic analyses of the WT, kaku4 mutant, *KAKU4*-complementation, and *KAKU4*-overexpression lines during leaf senescence.** (A) T-DNA insertion position of the *kaku4* mutant line (SALK_076754). (B) Constructs used for transformation of the *KAKU4*-complementation and *KAKU4*-overexpression lines. (C) Real-time RT-PCR analyses of the expression levels of *KAKU4* in the rosette leaves of the WT, *kaku4* mutant, *KAKU4*-complementation (*35S::KAKU4*/*kaku4-1* and *35S::KAKU4*/*kaku4-2*) and *KAKU4*-overexpression (*35S::KAKU*4/WT-1 and *35S::KAKU4/WT-2*) lines. (D) Rosette leaves of 4th-week WT, the *kaku4* mutant, *KAKU4*-complementation, and *KAKU4*-overexpression lines. (E) Leaf-senescence phenotype of the 6th-week WT, *kaku4* mutant, *KAKU4*-complementation, and *KAKU4*-overexpression lines. (F) Chlorophyll content in the 6th-week rosette leaves of WT, *kaku4* mutant, *KAKU4*-complementation, and *KAKU4*-overexpression lines. Significant differences between the mutant, transgenic line, and WT were determined according to Student’s t-test. *P-value ≤ 0.05, **P-value ≤ 0.01, ***P-value ≤ 0.001.

**Supplementary Figure S2. Phenotypic analyses of the WT and kaku4 mutants during leaf senescence.**  (A) Mutation sites in *KAKU4* modified by the CRISPR-Cas9 genome editing. Schema of *KAKU4* gene structure and the mutation sites of *kaku4-03*^Cas9^, *kaku4-04*^Cas9^, and *kaku4-05*^Cas9^. Boxes and lines in the diagram indicate exons and introns, respectively. Gray boxes indicate mutated parts in the mutants. (B) Rosette leaves of 3th-week *kaku4* mutant and WT during leaf senescence. (C) Plants of 6th-week *kaku4* mutant and WT during leaf senescence. (D) Rosette leaves of 7th-week *kaku4* mutant and WT during leaf senescence. (E) Chlorophyll content in the 7th-week rosette leaves of the *kaku4* mutants and WT.

**Supplementary Figure S3. Western blot detection of the H3K27me3 mark in *kaku4* mutant and WT.** Comparison between WT and *kaku4* mutant revealed reduction of H3K27me3-signal in the mutant. Detection of histone H3 was used a loading control. The Western blot with the full-length blot images.

## Supplementary Tables

**Supplementary Table S1.** Statistical summary of RNA-sequencing libraries. (see separate Word file)

**Supplementary Table S2.** Differentially expressed genes between the *kaku4* mutant and WT. (see separate Excel file)

**Supplementary Table S3.** The enriched GO terms for differentially expressed genes between the *kaku4* mutant and WT. (see separate Excel file)

**Supplementary Table S4.** List of primers used in this work. (see separate Word file)

**Supplementary Table S5.** GSEA analyses for differentially expressed genes between the *kaku4* mutant and WT. (see separate Excel file)

**Supplementary Table S6.** Overlap upregulated DEGs in *kaku4* mutant and *crwn1*, *crwn4*, *crwn1crwn4*, *pnet2abc*, *gbpl3*, and *ref6* mutants, and overlapping genes between upregulated DEGs in *kaku4* mutant and SARD1 target genes. (see separate Excel file)

**Supplementary Table S7.** Statistical summary of ChIP-sequencing libraries. (see separate Word file)

**Supplementary Table S8.** Genes associated with the H3K27me3 enriched regions in the *kaku*4 mutant or WT. (see separate Excel file)

**Supplementary Table S9.** Differential H3K27me3 related genes between the *kaku4* mutant and WT. (see separate Excel file)

**Supplementary Table S10.** Genes with decreased H3K27me3 deposition and upregulated in the *kaku4* mutant. (see separate Excel file)

**Supplemental Table S11.** One-way ANOVA results for comparisons in Figure 1. (see separate Word file)
