# Supplementary figures and images for: KAKU4 regulates leaf senescence through modulation of H3K27me3 deposition in the Arabidopsis genome
Source: BMC Plant Biol. 2024 Mar 7;24:177. doi: 10.1186/s12870-024-04860-9 (PMC10919013; doi:10.1186/s12870-024-04860-9)

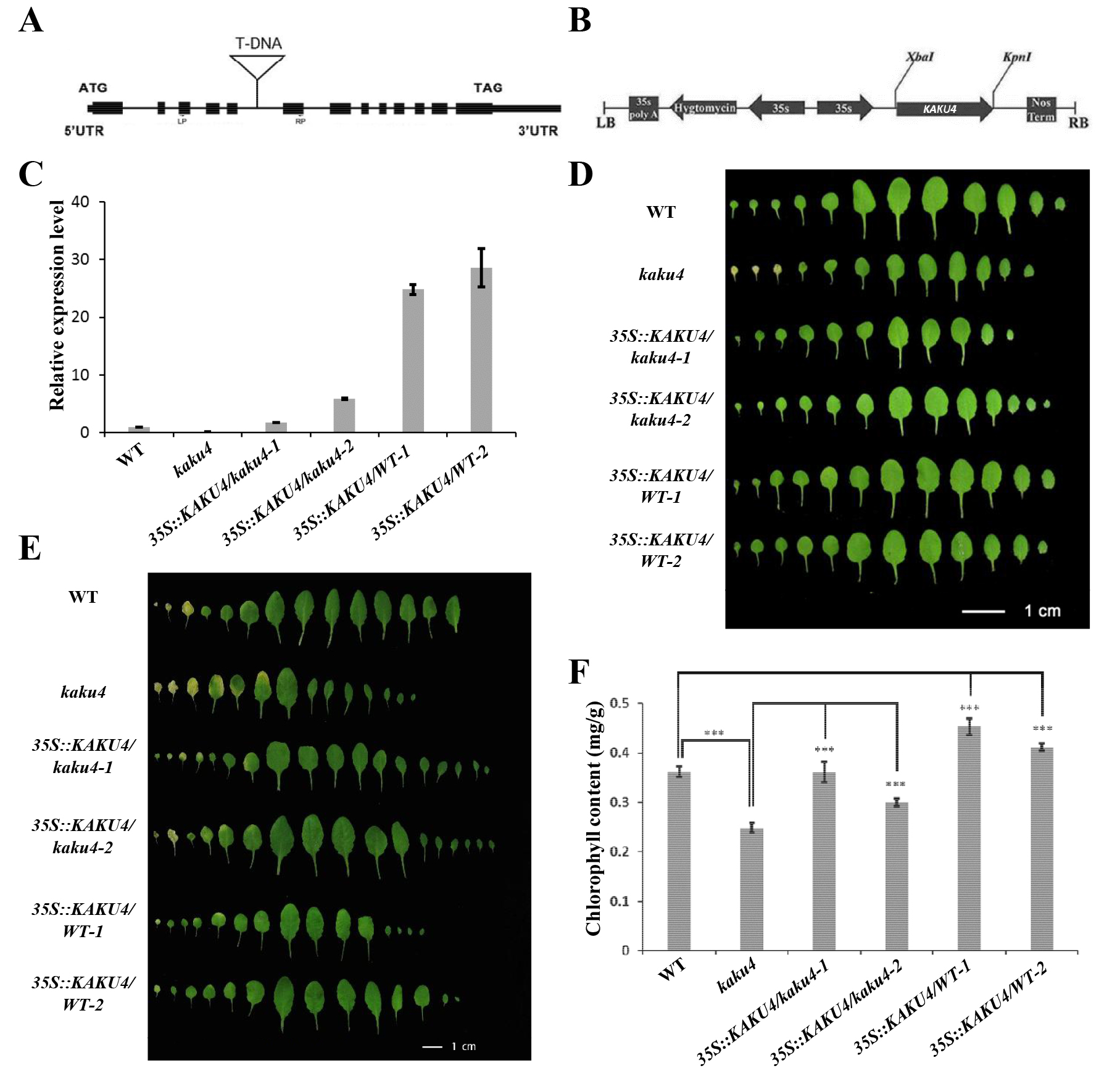

Supplement: Supplementary file 2 — Supplementary Material 2 [file 12870_2024_4860_MOESM2_ESM.jpg]

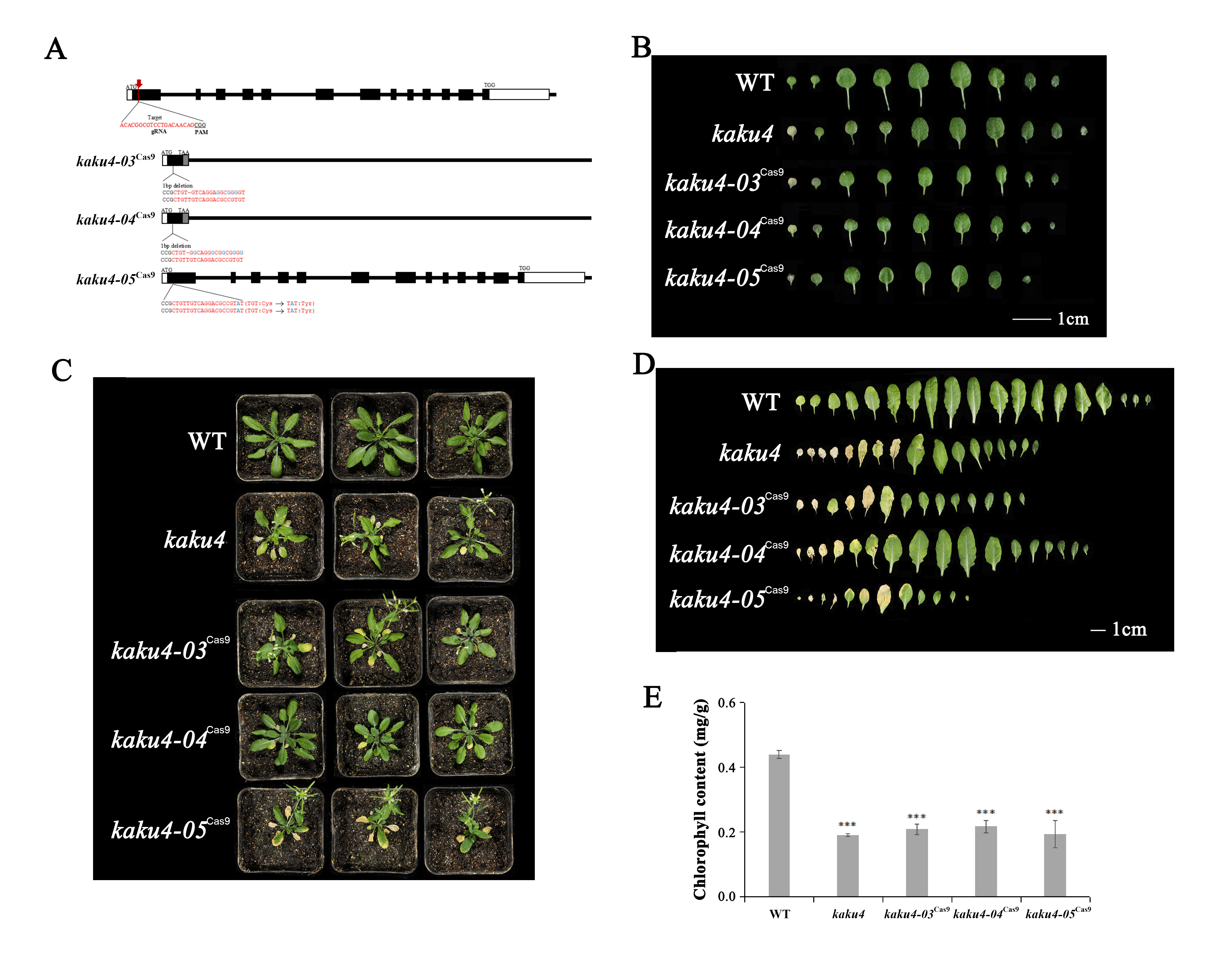

Supplement: Supplementary file 3 — Supplementary Material 3 [file 12870_2024_4860_MOESM3_ESM.jpg]

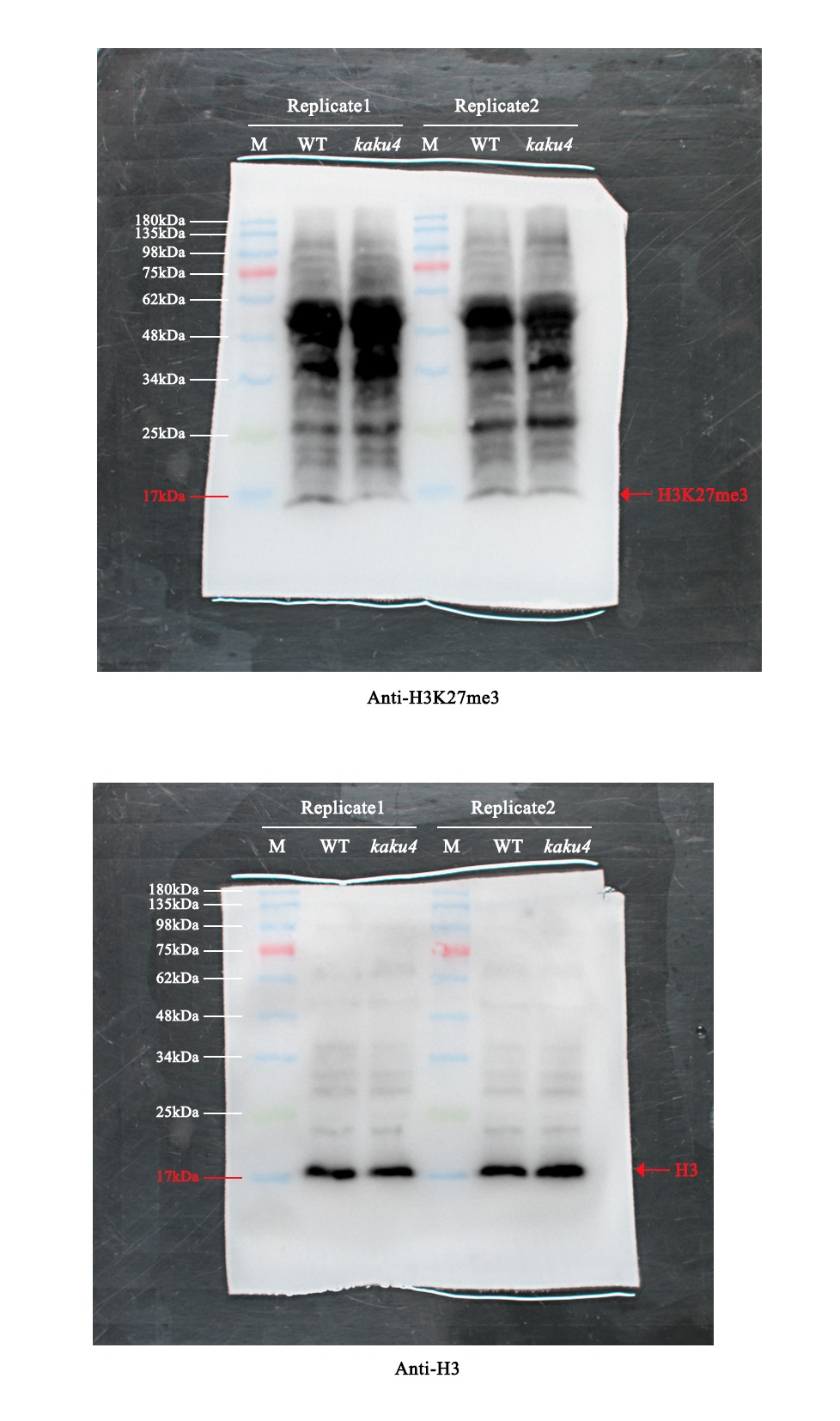

Supplement: Supplementary file 4 — Supplementary Material 4 [file 12870_2024_4860_MOESM4_ESM.jpg]
