# Supplementary material for: KAKU4 regulates leaf senescence through modulation of H3K27me3 deposition in the Arabidopsis genome
Source: BMC Plant Biol. 2024 Mar 7;24:177. doi: 10.1186/s12870-024-04860-9 (PMC10919013; doi:10.1186/s12870-024-04860-9)
Supplement: Supplementary file 5 — Supplementary Material 5 [file 12870_2024_4860_MOESM5_ESM.docx]

**Supplementary Table 1 | Statistical summary of RNA-sequencing libraries.**

| **Sample** | **Total reads** | **Mapped Reads** | **Mapping rate** | **Properly paired** |
| --- | --- | --- | --- | --- |
| *kaku4* rep1 | 47,105,856 | 44,244,959 | 93.90% | 89.50% |
| *kaku4* rep2 | 50,064,746 | 47,405,937 | 94.70% | 91.10% |
| *kaku4* rep3 | 65,531,118 | 61,068,050 | 93.20% | 89.60% |
| WT rep1 | 46,778,646 | 42,658,803 | 91.20% | 86.80% |
| WT rep2 | 67,171,746 | 62,429,146 | 92.90% | 89.00% |
| WT rep3 | 60,059,822 | 56,581,138 | 94.20% | 90.30% |
