# Supplementary material for: KAKU4 regulates leaf senescence through modulation of H3K27me3 deposition in the Arabidopsis genome
Source: BMC Plant Biol. 2024 Mar 7;24:177. doi: 10.1186/s12870-024-04860-9 (PMC10919013; doi:10.1186/s12870-024-04860-9)
Supplement: Supplementary file 8 — Supplementary Material 8 [file 12870_2024_4860_MOESM8_ESM.docx]

**Supplementary Table 4 | List of primers used in this work**

| **Primer name** | **Sequence (5'-3')** | **Application** |
| --- | --- | --- |
| 25S_FP | CAGTACGAATACGAACCGTG | ChIP-qPCR |
| 25S_RP | CAATGATAGGAAGAGCCGAC | ChIP-qPCR |
| PR2-FP | GGAGGCGAGACGTTCAAGAT | ChIP-qPCR |
| PR2-RP | ATGCTAGGCGATACCTTGCC | ChIP-qPCR |
| SAG113-FP | CGCCGGAAACAAACTTAAACCT | ChIP-qPCR |
| SAG113-RP | GGCTGAGATTTGTTACGAGAACG | ChIP-qPCR |
| MC2-FP | GACTATCACCGCTCGCTTCT | ChIP-qPCR |
| MC2-RP | ACCTTCTCCGTACACTCACG | ChIP-qPCR |
| SARD1-FP | CGCTACTCCCTTTCGCAACT | ChIP-qPCR |
| SARD1-RP | TGCCGGAGAAGTAAGTGTGA | ChIP-qPCR |
| PHT3-2-FP | CCGGCGAGTACATTTCCACT | ChIP-qPCR |
| PHT3-2-RP | GTCTTTTTCAGGCGACGACC | ChIP-qPCR |
| SALK_076754-LP | ACCAAGCATTCAACGACTCAG | Genotyping *kaku4* |
| SALK_076754-RP | GGATGAGGAAAAGTTTCCAGC | Genotyping *kaku4* |
| LB | GCGTGGACCGCTTGCTGCAACT | Genotyping *kaku4* |
| KAKU4_FP | GCTCTAGAATGGATTCCGTCTCCGGTTA | cDNA PCR |
| KAKU4_RP | GGGGTACCTTATTTGGCCCGTCCTT | cDNA PCR |
| ka-ex1_cas_F2 | CAACgAACTTCTCCCTCCTCC | Genotyping *kaku4-cas9* |
| ka-ex1_cas_R2 | ACACAACAAAGCTGGTTGGTATTC | Genotyping *kaku4-cas9* |
| 18S rRNA_F | CGGCTACCACATCCAAGGAA | Real-time RT-PCR |
| 18S rRNA_R | TGTCACTACCTCCCCGTGTCA | Real-time RT-PCR |
| kaku4Q-FP | GCTCCTCAAAGTGGAACTGC | Real-time RT-PCR |
| kaku4Q-RP | CCAAGACAGCATTTGTGGTG | Real-time RT-PCR |
| REF6Q-FP | AACCAGGTCAACAGCCAAAC | Real-time RT-PCR |
| REF6Q-RP | TTCTTGCAAAAAGCGACCTT | Real-time RT-PCR |
| SG113Q-FP | CTAGACCGGATTCAAGCAGC | Real-time RT-PCR |
| SG113Q-RP | GGCTCGTGACATTGCAAGTA | Real-time RT-PCR |
| SAG12Q-FP | TGATTGCGACACAAACGATT | Real-time RT-PCR |
| SAG12Q-RP | TTGACTCAGTTGTCAAGCCG | Real-time RT-PCR |
| SARD1Q-FP | TTGATGACTCAAACAAGCCG | Real-time RT-PCR |
| SARD1Q-RP | CGAAGGAAAATCTCCGTGAA | Real-time RT-PCR |
| WRKY33Q-FP | GGAGAGAGCATCACACGACA | Real-time RT-PCR |
| WRKY33Q-RP | GTGCTCTGTTTGTGGCGTAA | Real-time RT-PCR |
| EIN2Q-FP | GGGGGAGCCACTAATTTCAT | Real-time RT-PCR |
| EIN2Q-RP | TGAATGCACCAAACTCCAAA | Real-time RT-PCR |
| NCED2Q-FP | GGTTAGGAAGAAAAACCCGG | Real-time RT-PCR |
| NCED2Q-RP | ATCTCACCGGTGCAAAGATC | Real-time RT-PCR |
| RD29BQ-FP | TTCTTGGCTCGGTGGTAAAC | Real-time RT-PCR |
| RD29BQ-RP | ATCCGAAAACCCCATAGTCC | Real-time RT-PCR |
| CYP94B3Q-FP | TTCTGAACCGGGGAGTACAC | Real-time RT-PCR |
| CYP94B3Q-RP | TTTCCCTACACAAACCCTCG | Real-time RT-PCR |
| LOX4Q-FP | GCTCCAAGCCTGGTACTCTG | Real-time RT-PCR |
| LOX4Q-RP | GGTGATGACGGAGACGAGAT | Real-time RT-PCR |
| LOX1Q-FP | AAAGTCAGAACCATGGTGGC | Real-time RT-PCR |
| LOX1Q-RP | ACAGCTGCATGAAGAGCAGA | Real-time RT-PCR |
| NAPQ-FP | TTACATGGGACCCGTCTCTC | Real-time RT-PCR |
| NAPQ-RP | CCGAACCAACTAGACTCCGA | Real-time RT-PCR |
| ALD1Q-FP | CGGCTTGGTTGGTCTATCAT | Real-time RT-PCR |
| ALD1Q-RP | CGTTGAAGGAAGTGGTCACA | Real-time RT-PCR |
| BAP2Q-FP | CCTCAATACCAACAAGCCGT | Real-time RT-PCR |
| BAP2Q-RP | GTATAGGCCATCAGCGGGTA | Real-time RT-PCR |
| PR1Q-FP | CATACACTCTGGTGGGCCTT | Real-time RT-PCR |
| PR1Q-RP | TCGCTAACCCACATGTTCAC | Real-time RT-PCR |
| PR2Q-FP | CAGGGCTTGAAGTCAAGGTC | Real-time RT-PCR |
| PR2Q-RP | TCATCCCTGAACCTTCCTTG | Real-time RT-PCR |
