# Supplementary material for: KAKU4 regulates leaf senescence through modulation of H3K27me3 deposition in the Arabidopsis genome
Source: BMC Plant Biol. 2024 Mar 7;24:177. doi: 10.1186/s12870-024-04860-9 (PMC10919013; doi:10.1186/s12870-024-04860-9)
Supplement: Supplementary file 11 — Supplementary Material 11 [file 12870_2024_4860_MOESM11_ESM.docx]

**Supplementary Table 7 | Statistical summary of ChIP-sequencing libraries.**

| **Sample** | **Total reads** | **Mapped reads** | **Mapping rate** | **No. of Peaks** | **No. of genes related peaks** |
| --- | --- | --- | --- | --- | --- |
| *kaku4* | 21,397,353 | 20,382,644 | 95.26% | 3,748 | 4,560 |
| WT | 24,145,497 | 22,487,409 | 93.13% | 4,695 | 5,797 |
