# Supplementary material for: KAKU4 regulates leaf senescence through modulation of H3K27me3 deposition in the Arabidopsis genome
Source: BMC Plant Biol. 2024 Mar 7;24:177. doi: 10.1186/s12870-024-04860-9 (PMC10919013; doi:10.1186/s12870-024-04860-9)
Supplement: Supplementary file 15 — Supplementary Material 15 [file 12870_2024_4860_MOESM15_ESM.docx]

**Supplemental Table S11 | One-way ANOVA** **results for comparisons in Figure 1**

| **Comparison** |  | **df** | **SS** | **MS** | **F** | **P-value** |
| --- | --- | --- | --- | --- | --- | --- |
| **Figure 1C:**  **chlorophyll content** | Genotype | 1 | 0.713414 | 0.713414 | 58.59254 | **1.93E-08** |
|  | error | 29 | 0.353099 | 0.012176 |  |  |
|  | Total | 30 | 1.066513 |  |  |  |
| **Figure 1D:**  **ion leakage** | Genotype | 1 | 0.119031 | 0.119031 | 47.27037 | **2.37E-04** |
|  | error | 7 | 0.017627 | 0.002518 |  |  |
|  | Total | 8 | 0.136658 |  |  |  |
| **Figure 1E:**  **H_2_O_2_ content** | Genotype | 1 | 0.009572 | 0.009572 | 249.4681 | **9.39E-05** |
|  | error | 4 | 0.000153 | 3.837E-05 |  |  |
|  | Total | 5 | 0.009726 |  |  |  |
| **Figure 1F:**  **SA content** | Genotype | 1 | 38877.54 | 38877.54 | 564.4339 | **1.86E-05** |
|  | error | 4 | 275.5153 | 68.87882 |  |  |
|  | Total | 5 | 39153.06 |  |  |  |
| **Figure 1G:**  **JA content** | Genotype | 1 | 23216.56 | 23216.56 | 280.2651 | **7.46E-05** |
|  | error | 4 | 331.3514 | 82.83785 |  |  |
|  | Total | 5 | 23547.91 |  |  |  |
| **Figure 1H:**  **ABA content** | Genotype | 1 | 361.3795 | 361.3795 | 279.1429 | **7.52E-05** |
|  | error | 4 | 5.178416 | 1.294604 |  |  |
|  | Total | 5 | 366.5579 |  |  |  |

df: degree of freedom; SS: stdev square; MS: mean square

Bonferroni-adjusted (alpha=0.05) is 0.0167
